# Understanding the physical mechanism of intrinsic noise inside viscous isotropic solids

**DOI:** 10.1038/s41598-022-20228-1

**Published:** 2022-09-23

**Authors:** Lin Fa, Yimei Wang, Hong Gong, Dongning Liu, Jing Jiang, Lili Li, Jifeng Liang, Hao Sun, Yandong Zhang, Meishan Zhao

**Affiliations:** 1 School of Electronic Engineering, Xi’an University of Posts and Telecommunications, Xi’an, 710121 Shaanxi China; 2 School of Information Engineering, Xi’an Fanyi University, Xi’an, 710105 Shaanxi China; 3 James Franck Institute and Department of Chemistry, The University of Chicago, Chicago, IL 60637 USA

**Keywords:** Physics, Applied physics, Acoustics

## Abstract

We report acoustic impulse-response and system function of particle vibration inside viscous, dense solids and explain the physical mechanism of intrinsic-noise generation. With an external disturbance of a harmonic force acting on particles inside viscous solid media, the system of particle vibration goes through a gradual transition from a static state to a steady harmonic vibrational state. Based on the damped oscillator model, the transition frequency spectrum resembles the intrinsic noise generated by vibrating particles in viscous isotropic solids, which delivers a crucial understanding for applications to invert stratum characteristics around the drilled oil well and its abnormal geological structure.

## Introduction

Different solids are of various degrees of density and viscosity. There is a certain degree of viscosity for all solid media in nature. When a harmonic force acts on particles inside the solid media, there is a transition process of the particle's motion from a static state to a steady harmonic vibrational state induced by the inertia of the particle and the viscosity of the medium. Naturally, the particles in a solid generate intrinsic noise under external disturbance. The frequency spectrum corresponding to the transient process of the particle motion mimics the intrinsic noise generated by the particle inside viscous solids.

Intrinsic noise inside solids has been studied extensively for applications in various fields. Huet et al. investigated the contribution of viscosity to the generation and scattering of entropy noise in nozzles^1^. Hoffmann et al. studied the effect of damping on mode coupling instability in friction-induced vibration^2^. Zhu et al. further developed a flow-acoustic splitting method for predicting flow-acoustic noise by introducing a high-order finite-difference scheme^3^. Dragonetti et al. studied the statistical characteristics concerning the parameters of noise propagating in a solid medium, considering both the external and internal acoustic fields of a box^4^. Based on frequency domain analysis, Michael et al. described a frequency domain technique for analyzing intrinsic noise within negatively autoregulated gene circuits^5^. Ramaswamy et al. studied intrinsic frequency spectrum noise affecting mesoscopic oscillatory chemical reactions^6^. Alex et al. introduce a mathematical framework that extends classical extrinsic–intrinsic noise analysis^7^. Jangir et al. studied the effect of stochasticity inherent to biochemical reactions (intrinsic noise) and variability in cellular states (extrinsic noise), degrading information transmitted through signaling networks^8^. Villegas et al. investigated intrinsic noise and deviations from criticality in Boolean gene-regulatory networks^9^. Hong et al. pointed out that acoustic stress and wave resonance play a crucial role in plasma bubbles, and relevant acoustic studies offer new ways to achieve sustainable chemistry^10^. Kittmann et al. performed analyses of wideband low noise love wave magnetic field sensor system^11^. On the piezoelectric transient process from a stationary state to a vibration state, Piquette's theoretical and experimental research results showed that when a piezoelectric transducer was excited by a sinusoidal voltage signal, there was indeed a radiated acoustic signal transient process^12,13^. Fa et al. reported transient responses of radially polarized thin spherical shell transducers and radially and tangentially polarized piezoelectric thin circular tubes. They derived the analytical expressions of the electrical-acoustic impulse responses and system functions, then concluded that the convolution of the excitation voltage signal and electric-acoustic impulse-response would be the acoustic signals radiated by the transducers^14–18^. Still, there is more work to be done on classifying noises, intrinsic or external, and explaining the physical mechanism of generating intrinsic noise inside a viscous, dense solid and its application.

The acoustic signal measured by a near-bit noise-logging tool contains intrinsic and external noises. Unlike extrinsic noise, intrinsic noise arises from viscous solids' physical and geometrical properties. In practice, we may extract the intrinsic noise from the measured acoustic signal, then use the intrinsic noise to invert the physical characteristics of the formation around the oil well. From there, we may determine whether it is an oil or gas reservoir, calculate the oil or gas content, and evaluate the condition of the oil well. The concept of intrinsic noise can also be extended to the field of underwater acoustics. The noise generated from underwater targets belongs to external noise. In the process of detecting targets, e.g., stealth ships and sunken submarines, it is vital to eliminate the intrinsic noise generated by the marine environment, extract the external noise generated by the navigation and engine of the ships, and use the measured noise signal to track in real time and accurately locate the underwater targets.

We report, in this paper, the newly derived acoustic impulse-response and system function for the particle vibration inside viscous isotropic solids. We classify the noises generated inside viscous solid media (either intrinsic or extrinsic), deliver the physical mechanism of generating intrinsic noise, and discuss its application in industry.

Based on the harmonic oscillator model, the established time-domain and *s*-domain equivalent mechanical networks provide us with the acoustic-impulse response and system function of the particle vibration from the residue theorem and the physical mechanism of intrinsic noise generation.

We selected several types of rocks as samples to show the effect of the physical parameters on the intrinsic noise, e.g., viscosity, stiffness coefficient, density, and others. Our results showed that the intrinsic noise extracted from the measured acoustic signals was feasible to invert the physical properties of viscous solid and anomalies in its internal structure.

## Results

We selected Anisotropic shale, Mesaverade sandstone (*M-*sandstone), and Mesaverade-calcareous sandstone (*C*-sandstone) as the solid samples to perform the analysis^19^. Table 1 shows the relevant physical parameters of these rocks, and in the following analysis, we perform calculations and consider the isotropic nature of these solid samples. Here the symbols for frictional resistance, mass, and equivalent-stubborn coefficient of vibration particles inside the solids are $R_{m} = a_{1} \eta_{11}$, $m = a_{2} \rho$, $k_{c} = a_{3} c_{11}$, respectively; $a_{1}$, $a_{2}$ and $a_{3}$ are scale factors detailed in the Supplemental material.

**Table 1:** Parameters of the selected isotropic rocks.

Rock medium	*v*_*p*_ (*m/s*)	*ρ* (*g*/*cm*^*3*^)	$c_{11}$(*N*/*m*)
*A*-shale	2745	2.340	1.76 × 10^10^
*M*-Sandstone	3368	2.500	2.84 × 10^10^
*C*-Sandstone	4231	2.370	4.24 × 10^10^

*v_*p*_ is the phase velocity of a longitudinal wave of VTI rock in symmetric-axis direction, measured by Thomsen^19^, i.e., short of VTI rock media's anisotropy; ρ, $c_{11}$ and $\eta_{11}$ are density, stiffness coefficient, and viscosity coefficient of rock, respectively.

**Figure 1:**
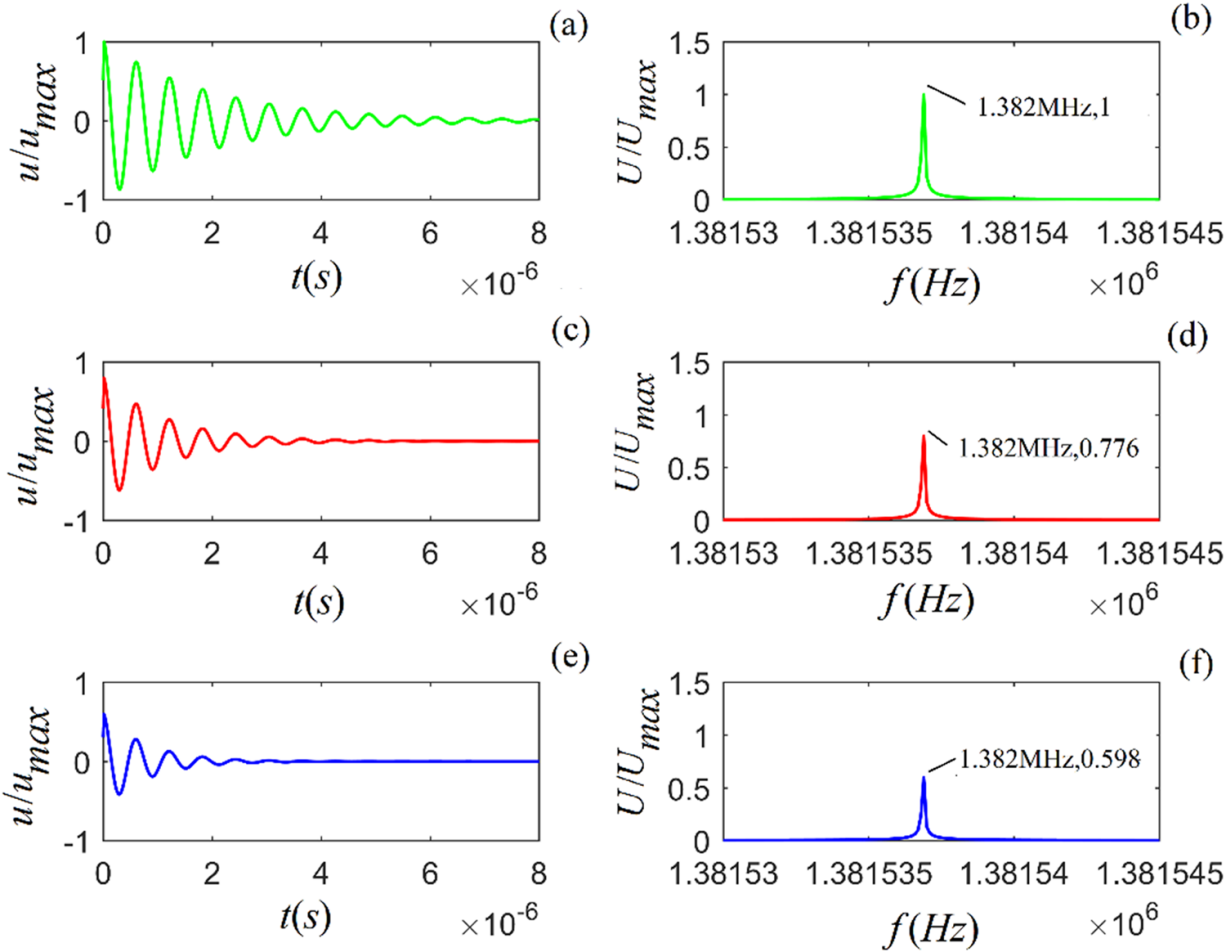
Acoustic impulse-response and amplitude spectrum of particle vibration in *A*-shale for three values of viscosity coefficient: $\eta_{11} { = }2 \times 10^{4}$, ${5} \times 10^{4}$ and ${8} \times 10^{4}$ $N\;s/m^{2}$) from top to bottom. (**a**), (**c**) and (**e**) are the time-domain waveforms, and (**b**), (**d**) and (**f**) are the corresponding amplitude spectra.

**Figure 2:**
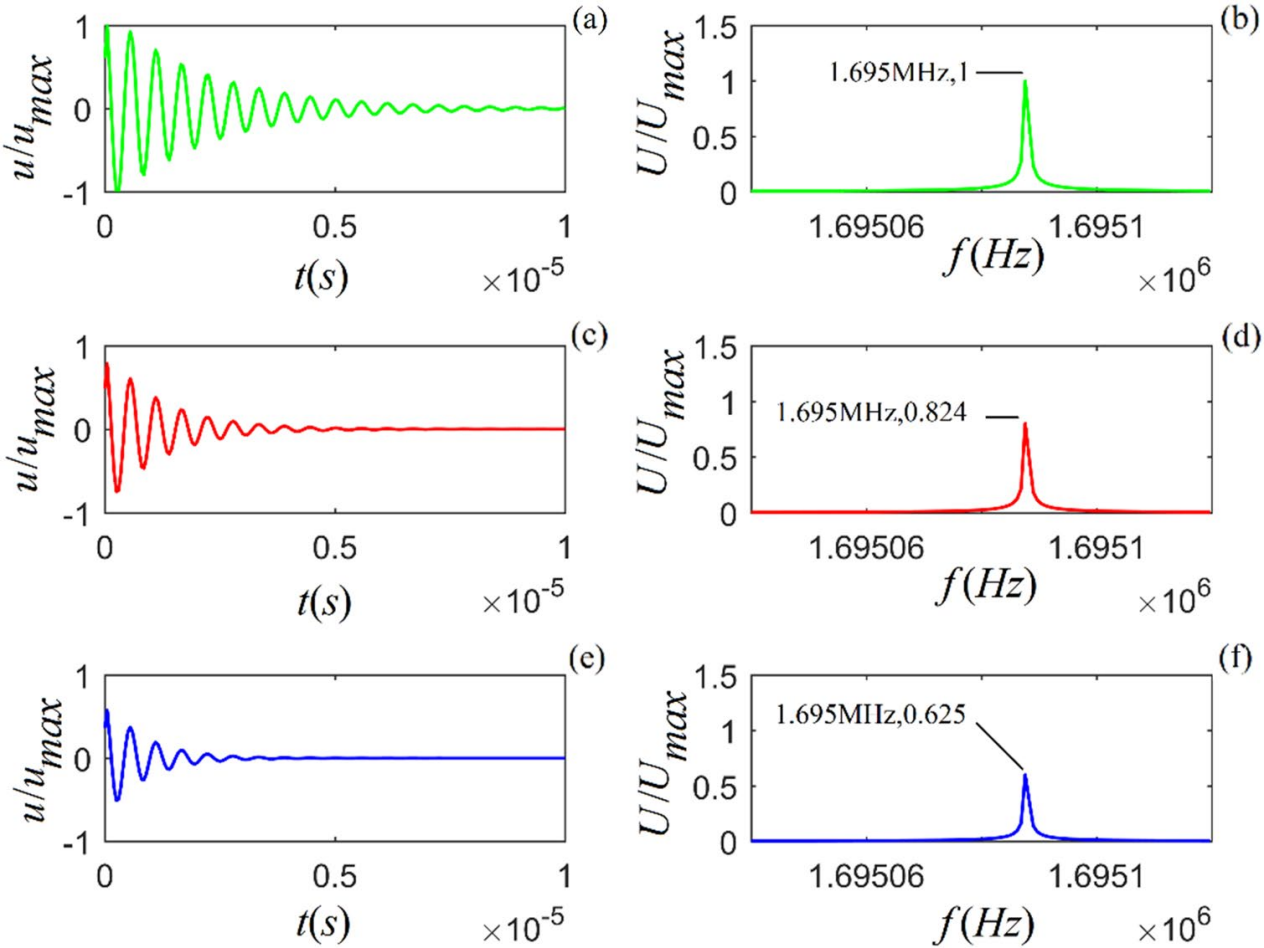
Acoustic impulse response and amplitude spectrum of particle vibration in *M*-sandstone for three values of viscosity coefficient: $\eta_{11} { = }2 \times 10^{4}$, ${5} \times 10^{4}$ and ${8} \times 10^{4}$ ($N\;s/m^{2}$) from top to bottom. (**a**), (**c**) and (**e**) are the time-domain waveforms, and (**b**), (**d**) and (**f**) are the corresponding amplitude spectra.

**Figure 3:**
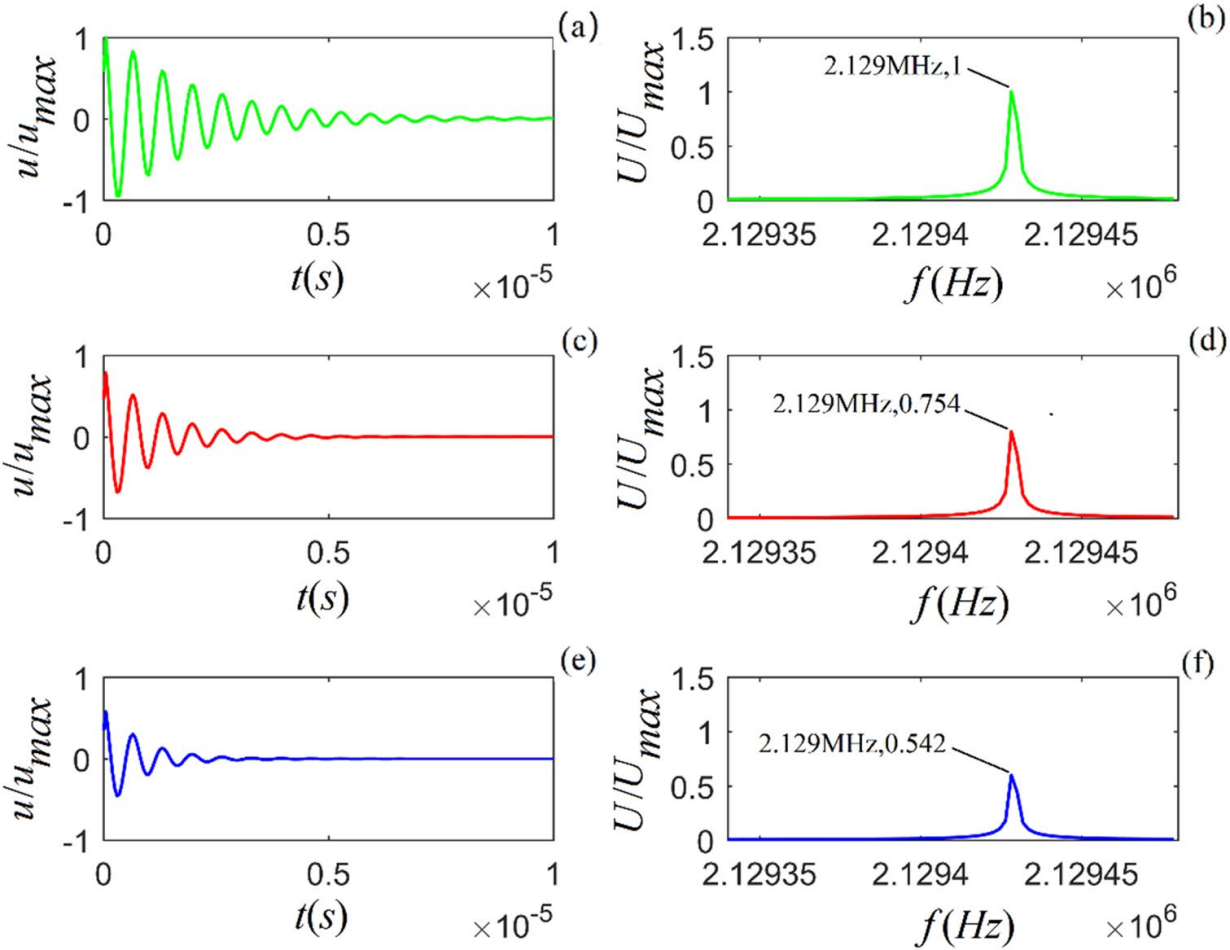
Acoustic impulse response and amplitude spectrum of particle vibration in *C*-sandstone for three values of viscosity coefficient: $\eta_{11} { = 4} \times 10^{4}$, $7 \times 10^{4}$ and $1 \times 10^{5}$ ($N\;s/m^{2}$) from top to bottom. (**a**), (**c**) and (**e**) are the time-domain waveforms, and (**b**), (**d**) and (**f**) are the corresponding amplitude spectra.

From Eqs. (12) and (13) (in the “Methods” section), the acoustic impulse-response and the corresponding amplitude spectra of particle vibration for the selected rock samples are calculated, as shown in Figs. 1–3.

Figures 1–3 show that for all three rock samples: (i) the amplitude of an acoustic impulse-response decays exponentially and periodically with time, and (ii) the corresponding amplitude spectrum increases with frequency until it reaches its maximum and then decreases with increasing frequency.

The frequency corresponding to the maximum amplitude spectrum ($f_{0}$) is called the center frequency of the particle vibration system. The calculation results also show that the viscosity coefficient ($\eta_{11}$) does not affect the center frequency of the system. However, it affects the magnitudes of the acoustic impulse response and corresponding amplitude spectrum: the larger the value of either $\eta_{11}$ or $a_{1}$, the faster the attenuation of the time-domain wave of the acoustic-impulse response and the smaller the corresponding amplitude spectrum. The physical parameters ($c_{11}$ and $a_{3}$) play a significant role in determining the center frequency, and the center frequency increases monotonically as either of these parameters increases. For the applied parameters, the center frequencies of shale, *M*-sandstone, and *C*-sandstone are 1.382 MHz, 1.695 MHz, and 2.129 MHz, respectively.

### Transient response of particle vibration under the influence of a harmonic force

**Figure 4:**
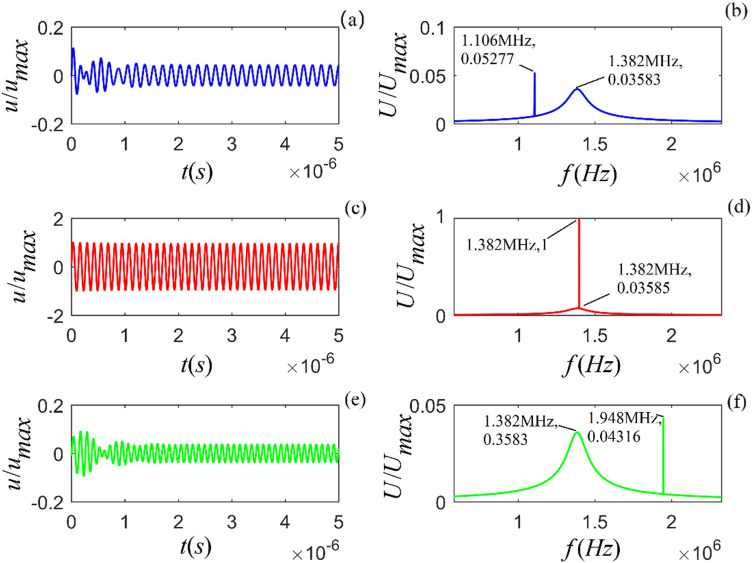
Time-domain waveform and amplitude spectrum of particle vibration for *A*-shale under the action of sinusoidal signal with selected frequencies ($f = 0.8f_{0}$, $1.0f_{0}$ and $1.2f_{0}$) from top to bottom. (**a**), (**c**) and (**d**) are the time-domain waveforms of particle vibration, respectively; (**b**), (**e**) and (**f**) are the corresponding amplitude spectra.

**Figure 5:**
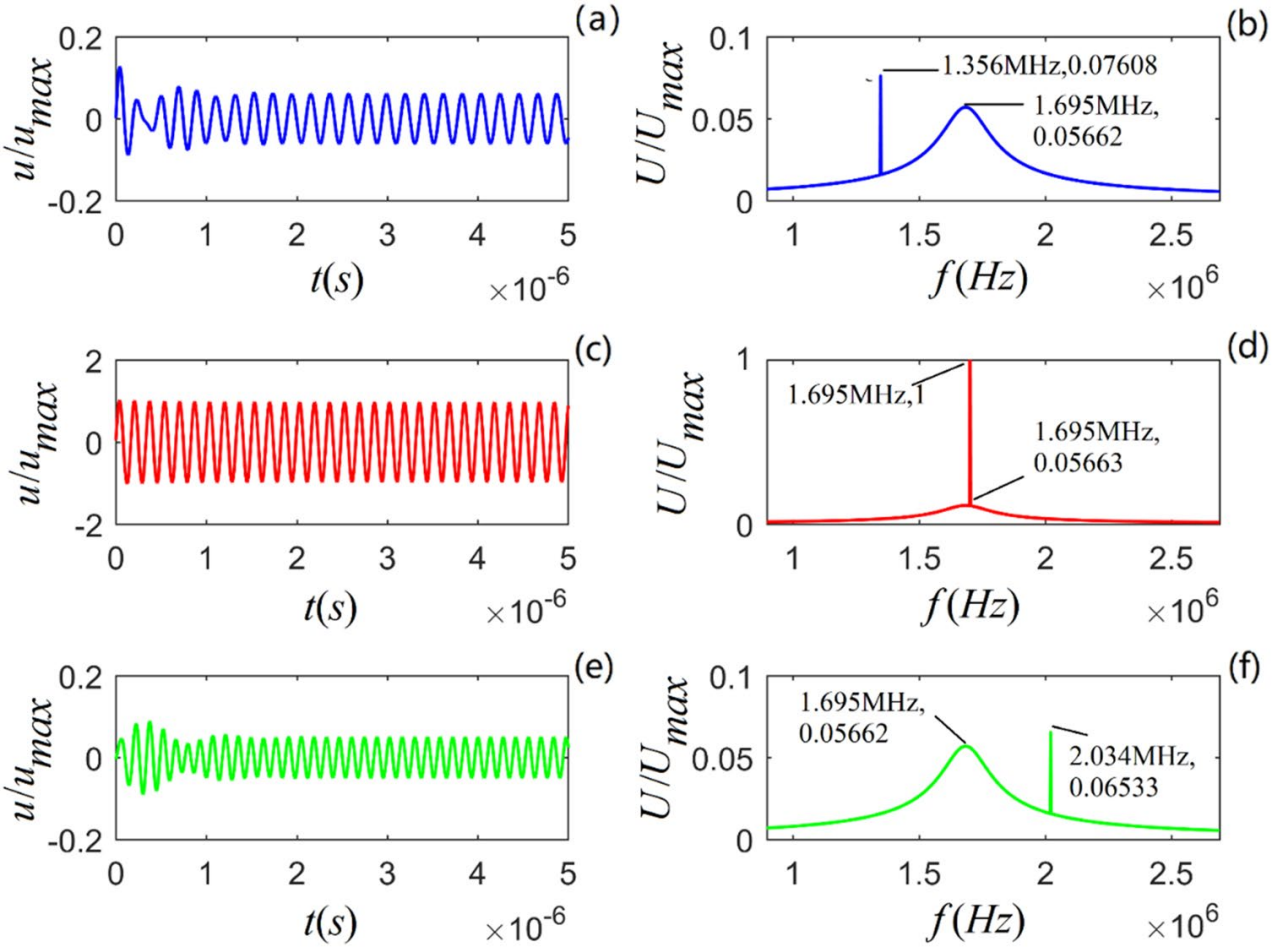
Time-domain waveform and amplitude spectrum of particle vibration for *M*-sandstone under the action of sinusoidal signal with selected frequencies ($f = 0.8f_{0}$, $1.0f_{0}$ and $1.2f_{0}$) from top to bottom. (**a**), (**c**) and (**d**) are the time-domain waveforms of particle vibration, respectively; (**b**), (**e**) and (**f**) are the corresponding amplitude spectra.

**Figure 6:**
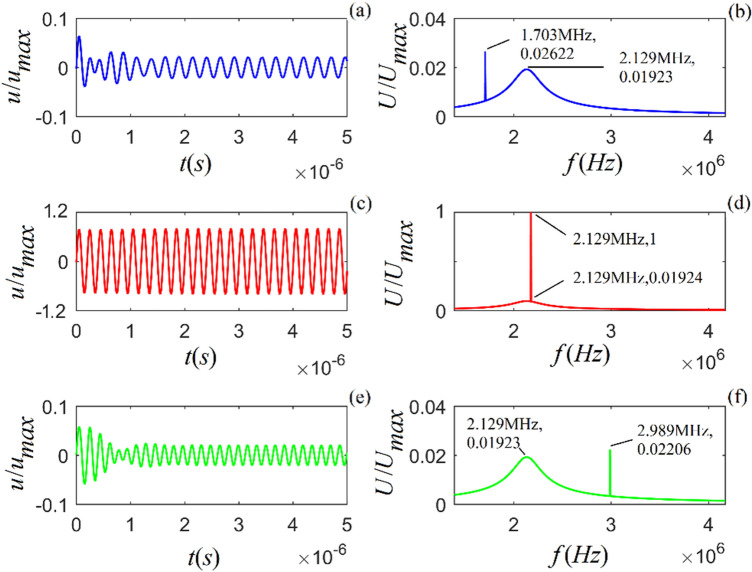
Time-domain waveform and amplitude spectrum of particle vibration system for *C*-sandstone under the action of a sinusoidal signal with selected frequencies ($f = 0.8f_{0}$, $1.0f_{0}$ and $1.2f_{0}$) from top to bottom. (**a**), (**c**) and (**d**) are the time-domain waveforms of particle vibration, respectively; (**b**), (**e**) and (**f**) are the corresponding amplitude spectra.

From Eq. (16) (in the “Methods” section) and its Fourier transform, applying sinusoidal signals with the selected frequencies ($f = 0.8f_{0}$, $1.0f_{0}$, and $1.2f_{0}$), the calculated time-domain waveform and amplitude spectrum for the rock samples are shown in Figs. 4–6.

Figures 4–6 show that (i) the time-domain wave of the particle vibration consists of two parts, i.e., a transient transition process and a steady harmonic vibration with the sinusoidal signal frequency; (ii) the amplitude spectrum also consists of two parts, i.e., a continuous smooth amplitude spectrum curve corresponding the transition process and an impulse with the sinusoidal signal frequency, which corresponds to steady-state harmonic vibration of the particle; (iii) the frequency spectrum corresponding to the transition process of particle vibration is the intrinsic-noise generated in viscous solid; (iv) the values of amplitude spectrum and waveform decrease when the frequency of harmonic force is far from the center frequency of particle vibration, and for this case, we can clearly recognize the intrinsic-noise determined by the physical property and internal structure of the viscous solid from the measured acoustic signal; (v) the amplitude spectrum and waveform of the particle vibration reach their maxima when the sinusoidal signal frequency is equal to the center frequency of particle vibration, and for this case, the frequency of the intrinsic-noise is aliased with the frequency of the harmonic force.

### Transient response of particle vibration under the action of a sinusoidal signal with muti-frequencies

In practice, the force acting on a system of particles in a viscous solid is usually a signal wavelet containing many frequency components. Let us select a gated sinusoidal signal wavelet for applications in the sample rocks^18^. The amplitude and window-width of the signal are 1.0 and 3.0 T, respectively, where "1.0" indicates that the amplitude of the gated harmonic force is 1, and T is a sine-cycle of a sinusoidal signal whose corresponding frequency can be $0.8f_{0}$, $1.0f_{0}$, and $1.2f_{0}$, respectively.

**Figure 7:**
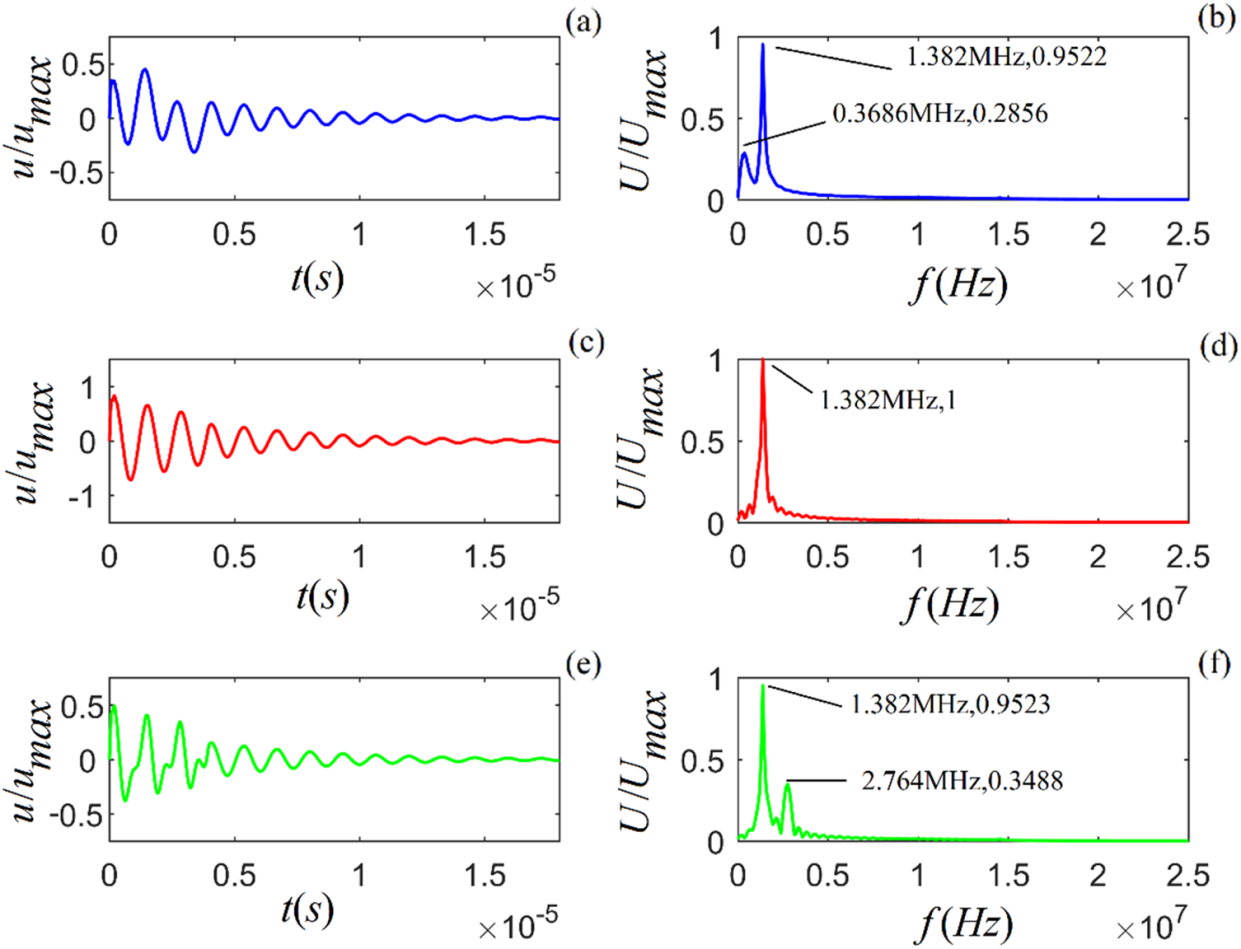
Time-domain waveform and amplitude spectrum of the particle vibration in *A*-shale. (**a**), (**c**) and (**e**) are for the cases of gated sinusoidal signal frequencies $0.8f_{0}$, $f_{0}$ and $1.2f_{0}$, respectively; (**b**), (**e**), and (**f**) are the corresponding amplitude spectra.

**Figure 8:**
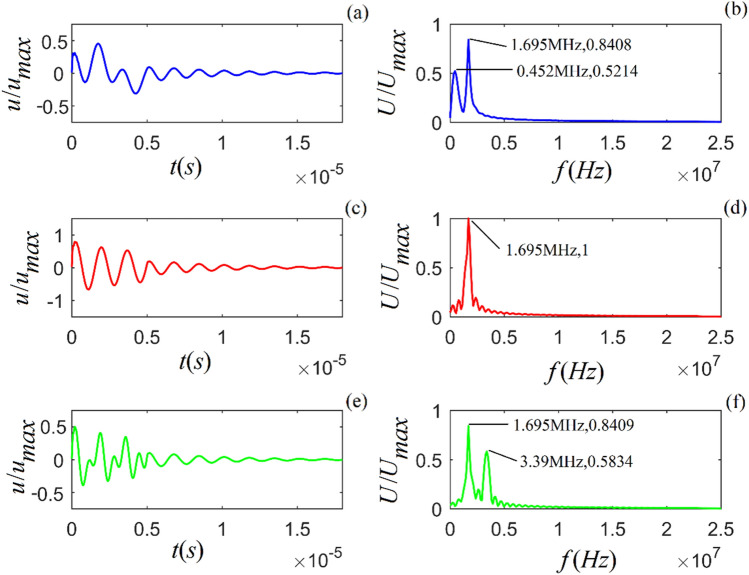
Time-domain waveform and amplitude spectrum of the particle vibration in the *M*-sandstone. (**a**), (**c**) and (**e**) are for the cases of gated sinusoidal signal frequencies $0.8f_{0}$, $f_{0}$ and $1.2f_{0}$ respectively; (**b**), (**e**), and (**f**) are the corresponding amplitude spectra.

**Figure 9:**
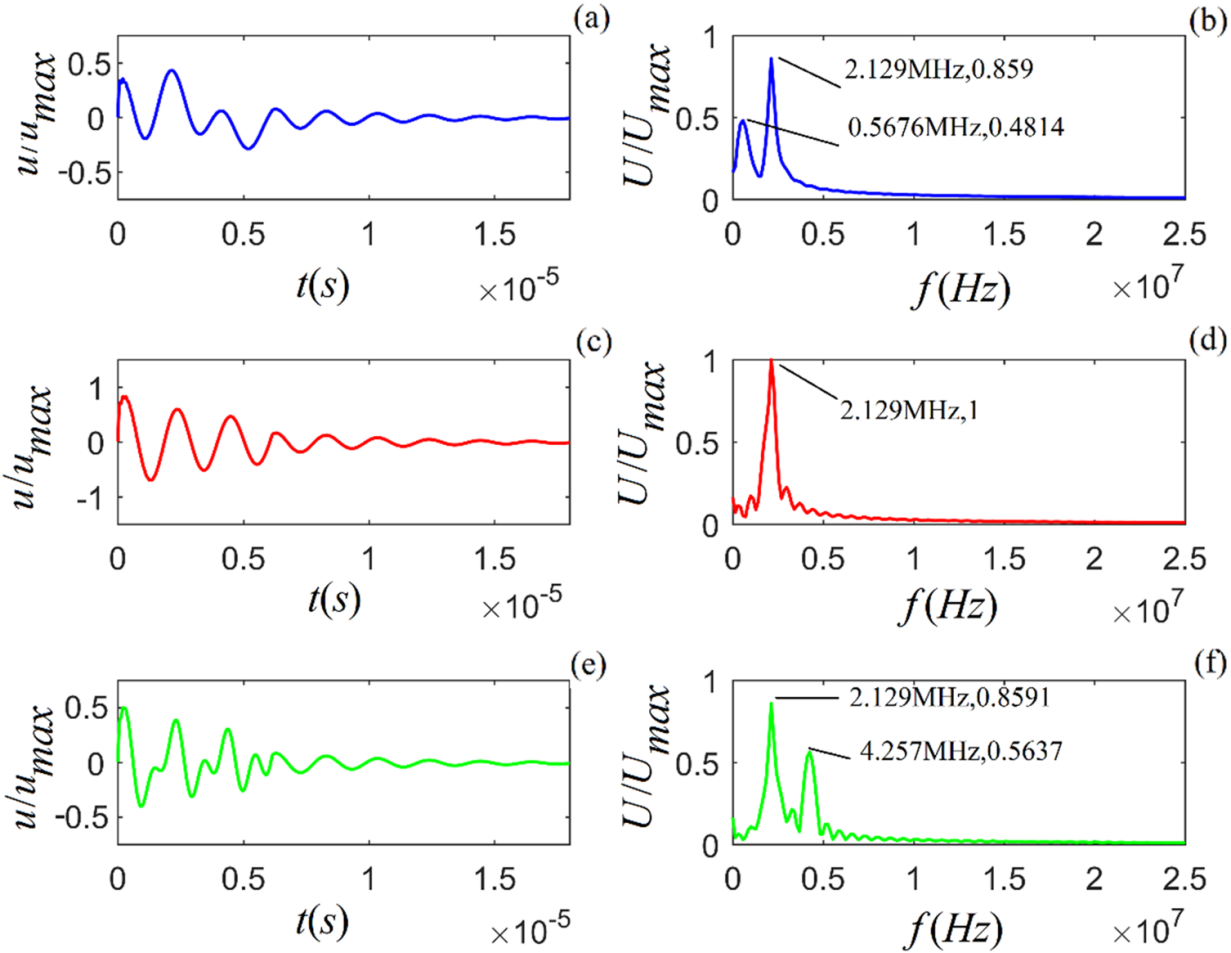
Time-domain waveform and amplitude spectrum of the particle vibration in the *C*-sandstone. (**a**), (**c**) and (**e**) are for the cases of gated sinusoidal signal frequencies $0.8f_{0}$, $f_{0}$ and $1.2f_{0}$, respectively; (**b**), (**e**), and (**f**) are the corresponding amplitude spectra.

Figures 7–9 illustrate the time-domain waves and amplitude spectra normalized by the corresponding maxima of the gated sinusoidal frequencies, which is also the center frequency ($f_{0}$).

From Figs. 4–9, we find that different sinusoidal signals acting on the particle may lead to distinct vibration states in each rock sample. The nature of the force would not influence the physical property of intrinsic noise, e.g., the center frequency. A harmonic force would yield more accurate information regarding intrinsic noise generated from the measured acoustic signals than using other external disturbances. Additionally, the amplitude spectrum in Figs. 4–6 includes an impulse due purely to the frequency of a harmonic force acting on the particle. Figures 7–9 contain continuous amplitude spectra corresponding to an external gated sinusoidal signal.

The measured acoustic signal from practical applications contains the frequency components of external disturbances, e.g., the force applied to the system of the particle vibration, external noise from the environment, and the intrinsic-noise corresponding to the transient process of particle vibration. The noise signal measured by a near-bit noise-logging tool contains the external noise generated by the drilling rig and includes the induced intrinsic noise. The characteristics of the intrinsic noise depend on the solid medium's physical properties, such as viscosity, stiffness coefficient, density, and more. We can invert the rocks' physical properties and internal structure from the intrinsic noise generated by particle vibration. For example, we may apply specific algorithms to extract the intrinsic noise from the measured noise signal and use its time and frequency domain properties to invert the physical and mechanical properties of the formation around the drilled oil well.

## Discussion

From calculation and analysis, we make the following concluding remarks. (i) The convolution of the sinusoidal signal acting on the system with the acoustic impulse response is suitable to describe the vibrational state of the particle. The acoustic-impulse response and system function reflect the inherent physical properties of viscous solid media, which helps analyze the physical phenomena generated by acoustic waves propagating inside the medium, e.g., the generation of intrinsic noise, acoustic attenuation, and dispersion.

(ii) Under the action of the harmonic force, the particle inside a solid medium has a transient process from a static state to a stable harmonic vibrational state. The vibrational spectrum corresponding to this transient process is the same as the spectrum of the intrinsic noise generated in the viscous solid. The physical parameters of the solid determine its inherent noise. Then, we can obtain the solid's physical properties and internal anomalies from its intrinsic noise.

(iii) Compared to the multi-frequency wavelet acting on the system of the viscous solid, a sinusoidal signal can separate the frequency spectrum of the intrinsic noise more accurately and efficiently from the measured acoustic signal.

(iv) The center frequency of the vibrational motion of particles inside a solid is related to the stiffness coefficient ($c_{11}$) and a scale factor ($a_{3}$). It is irrelevant to the viscosity coefficient ($\eta_{11}$), which only affects the duration of the transient process. The larger the viscosity coefficient (or the scale factor $a_{3}$), the shorter the transient process.

(v) With different external forces acting on a solid, the vibrational motion of the particle has a different transient process. The closer the sinusoidal signal frequency is to the center frequency, the greater the amplitude of the particle vibration.

(vi) The characteristics of the intrinsic noise in a measured acoustic signal are closely related to the physical characteristics of a solid sample, e.g., viscosity, stiffness coefficient, density, internal geometric structure (e.g., the existence of fractures), and more. By applying acoustic logging while drilling, we can use the intrinsic noise to obtain the physical properties of the formation around a drilled oil well and evaluate oil-well conditions. i.e., determine whether it contains fractures and if it is an oil or gas reservoir.

The physical mechanism of generating intrinsic noise in viscous solids described in this paper enhances our understanding of the new physical phenomena in acoustics. It provides us with the tools to deduce or simulate the intrinsic noise in viscous solids. In the subsequent process, we describe the experimentation methods, verify the physical mechanism of the intrinsic-noise generation, and develop the corresponding inversion interpretation processing software for the intrinsic noise. The theoretical results would lay the foundation for establishing new intrinsic-noise logging tools and provide us with a new scheme to convert academic research into industrial applications and products. Still, we would appreciate more practical experimental verification.

## Methods

### Physical model of particle vibration system in viscous solid medium

An electromagnetic wave is a substance without mass but energy and has propagation attenuation only if propagating in a medium with non-zero conductivity. However, a vibrating particle inside a viscous solid has both mass and energy. Due to the inertia of the particle and the resulting frictional force, there is a transient process for the particle from a static state to a stable harmonic vibration state when a harmonic force acts it. Therefore, the particle vibration contains the frequency component of the steady-state harmonic vibration and some frequency components of the transient process corresponding to the particle vibration. These are the frequency components of intrinsic noise generated in viscous solids. In the following, we analyze and discuss the vibration state of the particle in viscous solid and provide a new and insightful explanation of the physical mechanism of intrinsic-noise generation.

When a particle moves in a viscous fluid, it is subject to frictional resistance. The magnitude of this frictional force is related to the viscosity of the medium and its shape, size, and movement speed. For example, the frictional force of a small ball moving at a uniform speed in a viscous liquid is^20.^

1 $$f = 6\pi r\eta v = R_{0} v$$

There are several important parameters: *r* and *v* are the radius and the moving speed, respectively; $\eta$ is the viscosity coefficient of the medium; $R_{0} = 6\pi r\eta$ is frictional resistance. The frictional force experienced by the particle is proportional to the movement speed of the small ball, the geometric (shape and size) parameters $6\pi r$ and the viscosity $\eta$ of the liquid.

Analog to a small moving ball in a viscous fluid, we assume that the vibration particles in viscous, dense solids act like tiny balls. The frictional force experienced by a vibrating particle is proportional to its vibration velocity and the viscous coefficient of the solid. The direction of the frictional force is opposite to the moving direction of the particle.

**Figure 10:**
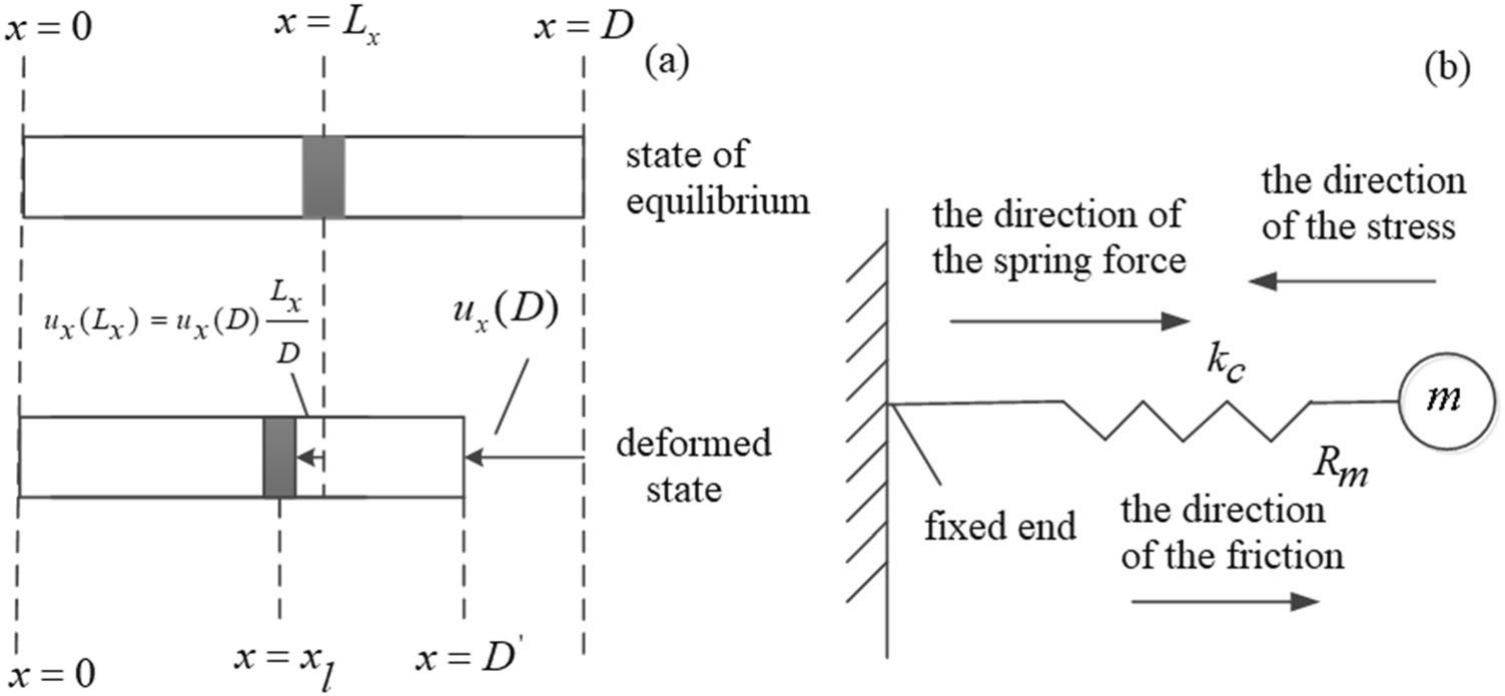
A model mechanical analog of particle damping vibrations corresponding to a longitudinal wave: (**a**) compression deformation of a uniform rod and (**b**) equivalent spring oscillator.

Elastic damping is quite complex, related to the type of strain of the solid caused by the particle vibration. The compressive strain corresponding to the longitudinal wave differs from the shear strain corresponding to the shear wave. For compressive strain, the size and density of the volume element will change. Figure 10(a) shows the compression deformation of a uniform rod. The compressive strain of the rod is equivalent to a spring oscillator for particle vibration corresponding to the longitudinal wave, as shown in Fig. 10(b). We assume that the spring's length ($l_{0}$) corresponds to the particle's equilibrium position, and the spring oscillator's instantaneous length is *l* during the vibration process. Also, the particle displacement is *u*, and its direction corresponds to the longitudinal wave, which is parallel to the horizontal direction.

It can be seen from Fig. 10 that the vibrating particles inside viscous solids are not only affected by the ideal microscopic "spring force" but also affected by frictional force. Assume that the frictional resistance of the particle with a spherical shape inside viscous solids is $R_{m}$. By analogy to the frictional resistance $R_{0}$ of the ball moving inside the viscous fluid described in formula (1), we can think that the magnitude $R_{m}$ is also proportional to the viscosity of the solid. The frictional force direction is always opposite to the moving direction of the particle,

2 $$f_{f} = - R_{m} \frac{du}{{dt}}$$

And the particle displacement corresponding to the longitudinal wave is

3 $$u = \, x = l - l_{0}$$

From Hooke's theorem, we can express the elastic force acting on the vibrating particle as

4 $$f_{k} = - k_{c} \Delta l = - k_{c} x = - ku$$

The elastic constant $k_{c}$ is the stubbornness coefficient of the spring oscillator.

### Impulse response and system function of particle vibration inside viscous solid media

When a harmonic force ($f_{a}$) acts on a particle inside viscous solid media, the equation of motion is given by

5 $$f_{a} = m\frac{{d^{2} u}}{{dt^{2} }} + R_{m} \frac{du}{{dt}} + k_{c} u$$

The physical quantities and physical parameters involved in Eq. (5) are the particle displacement (*u*(t)), particle displacement velocity (*v*(*t*) = *du*(*t*)/*dt*), mass (*m*) of the particle, the reciprocal of the stubbornness coefficient ($C_{m} = 1/k_{c}$), friction resistance (*R*_*m*_), the force *f*_*a*_ (*t*) in mechanical network, and they are analogized to the charge (*Q*(*t*)), the current (*I*(*t*)), the inductance (*L*), the capacitance (*C*), the resistance (*R*), and the voltage (*U*(*t*)) in the electrical network, respectively.

**Figure 11:**
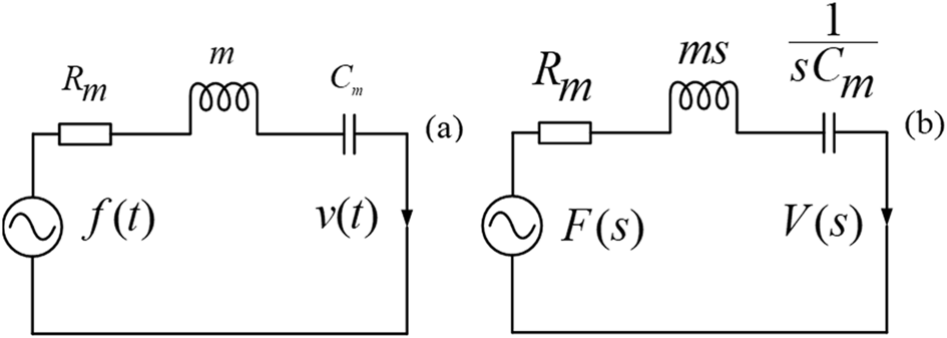
Equivalent mechanical network of the vibrational motion of the particle inside viscous solids. (**a**) time-domain; (**b**) *s-*domain.

From Eq. (5), we may obtain a time-domain network, as shown in Fig. 11(a), and the corresponding *s*-domain network in Fig. 11(b), where *V*(*s*) and *F*_*a*_(*s*) are the expressions of the particle displacement velocity and the force acting on the particle in the *s*-domain, respectively.

The particle displacement velocity, shown in Fig. 11(b), is in the *s*-domain,

6 $$V(s) = \frac{{F_{a} (s)}}{{R_{m} + ms + 1/C_{m} s}}$$

From Eq. (6), the system function of the mechanical network, i.e., the ratio of $V(s)$ to $F_{a} (s)$), is

7 $$H(s) = \frac{V(s)}{{F_{a} (s)}} = \frac{1}{{R_{m} + ms + 1/C_{m} s}} = \frac{{C_{m} s}}{{mC_{m} s^{2} + R_{m} C_{m} s + 1}}$$

Based on the residue theorem, the impulse response of the mechanical network in the time domain is given by

8 $$h(t) = \sum\limits_{j = 1}^{N} {{\text{Re}} s} \left[ {H(s_{j} )e^{{s_{j} t}} } \right]$$

where $N$ is the number of singularities, the number of roots of the denominator of Eq. (7), and $s_{j}$ is the *jth* singularity. The roots of the denominator of Eq. 7 are

9 $$s_{1,2} = \frac{{ - R_{m} C_{m} \pm \sqrt {(R_{m} C_{m} )^{2} - 4mC_{m} } }}{{2mC_{m} }}$$

The three cases of $(R_{m} C_{m} )^{2} > 4mC_{m}$, $(R_{m} C_{m} )^{2} = 4mC_{m}$, and $(R_{m} C_{m} )^{2} < 4mC_{m}$ are the following.

i) $(R_{m} C_{m} )^{2} > 4mC_{m}$: $s_{1,2}$ are the two different real roots. Solving Eq. (8) yields the solution.

10 $$h(t) = (A_{1} e^{ - \alpha t} + A_{2} e^{ - \beta t} ) \varepsilon (t)$$

In which,$$\beta = R_{m} /2m$$,$$\alpha = \left( {(R_{m} C_{m} )^{2} - 4mC_{m} } \right)^{1/2} /2mC_{m}$$$$A_{1} = C_{m} \beta /\left( {\beta - \alpha } \right)$$, $$A_{2} = - C_{m} \alpha /\left( {\beta - \alpha } \right)$$, and $$\varepsilon (t)$$ are unit step functions. Equation 10 indicates that the particle is in an overdamped state and is, obviously, not a reasonable solution for particle vibration.

ii) $(R_{m} C_{m} )^{2} = 4mC_{m}$: $s_{1,2}$ are two identical real roots with an equation of motion

11 $$h(t) = (A_{1} t + A_{2} )e^{ - \beta t} \varepsilon (t)$$

where $\alpha = 0$,$A_{1} = C_{m} \beta$ and $A_{2} = C_{m}$. Equation (11) indicates that the system is in a critical damped state and is not a reasonable solution.

iii) For $(R_{m} C_{m} )^{2} < 4mC_{m}$: $s_{1,2}$ are a pair of complex conjugate roots, and the impulse response is

12 $$h(t) = Ae^{ - \beta t} \cos (\omega_{d} t + \theta )\varepsilon (t)$$

where $A = C_{m} \left( {1 + \left( {\beta /\alpha } \right)^{2} } \right)^{1/2}$,$\omega_{d} = \left( {4mC_{m} - (R_{m} C_{m} )^{2} } \right)^{1/2} /2mC_{m}$,$\theta = \tan^{ - 1} \left( {\beta /\omega_{d} } \right)$. We define $\beta$ as the damping coefficient of particle vibration. The amplitude $A{\text{e}}^{ - \beta t}$ of the impulse response decays exponentially with time.

Equation (12) indicates that the particle system is in an under-damped mode with a solution corresponding to the actual physical meaning of "particle vibration." The absolute integrability of *h* (*t*) results in $s = i\omega$, so Eq. (7) is the system function of the particle vibration inside viscous solids, and we may revise it as

13 $$H(\omega ) = H(s)|_{s = i\omega } = \frac{{i\omega C_{m} }}{{ - mC_{m} \omega^{2} + iR_{m} C_{m} \omega + 1}}$$

Equations (12) and (13) show that the solid medium's physical properties determine the particle vibration's impulse response within the system. We infer that the physical properties of a viscous solid and its structural abnormity can be inverted by the intrinsic noise generated inside viscous solid media.

Equation (5) is the equation of the vibrational motion of the particle inside viscous solids when applying an external force $f_{a}$ to the particle. As a second-order differential equation with constant coefficients, its complete solution includes a general solution of the corresponding homogeneous equation and a special solution ($u = C_{m} f_{a}$) of an inhomogeneous equation.

From Eq. (5), its eigenequation is then

14 $$\lambda^{2} + \frac{{R_{m} }}{m}\lambda + \frac{1}{{mC_{m} }} = 0$$

which yields a general solution of the homogeneous equation

15 $$u_{g} = Ae^{ - \beta t} \cos (\omega_{d} t + \theta )\varepsilon (t)$$

The coefficient *(A)* and phase angle ($\theta$) in Eq. (15) will be determined by the initial conditions. If the force *f*_*a*_ acting on the particle inside viscous solids is a harmonic force, the general solution is then

16 $$u = u_{s} + u_{g} = f_{a} C_{m} + Ae^{ - \beta t} \cos (\omega_{d} t + \theta )$$

The first term describes the steady-state harmonic vibration of the particle. The second term does the transient process of particle vibration jointly determined by the harmonic force *f*_*a*_ and the inherent physical properties of the particle system.

## Supplementary Information

Supplementary Information.

## Data Availability

The data supporting the findings presented in this work are available from the corresponding author upon reasonable request.
