# Supplementary material for: Understanding the physical mechanism of intrinsic noise inside viscous isotropic solids
Source: Sci Rep. 2022 Sep 23;12:15878. doi: 10.1038/s41598-022-20228-1 (PMC9508097; doi:10.1038/s41598-022-20228-1)
Supplement: Supplementary file 1 — Supplementary Information. [file 41598_2022_20228_MOESM1_ESM.docx]

**Supplementary material for “Understanding the physical mechanism of intrinsic noise inside viscous isotropic solids”**

Lin Fa, Yimei Wang, Hong Gong, Dongning Liu, Jing Jiang, Lili Li, Jifeng Liang, Hao Sun, Yandong Zhang & Meishan Zhao

^1^ School of Electronic Engineering, Xi'an University of Posts and Telecommunications, Xi'an, Shaanxi 710121, China.

^2^ School of Information Engineering, Xi'an Fanyi University, Xi'an, Shaanxi 710105, China.

^3^ James Franck Institute and Department of Chemistry, The University of Chicago, Chicago, IL 60637, USA.

Correspondence and requests for materials should be addressed to L. F. and M. Z. (email: [faxiaoxue@126.com](mailto:faxiaoxue@126.com) and m-zhao@uchicago.edu).

**Description of the scale factors ($a_{1}$,$a_{2}$ and $a_{3}$).** Auld gave the damped elastic constitutive relation of viscous solid as follows^S1^

$\text{T}=c:\text{S}+\eta:\frac{\partial\text{S}}{\partial t}$ (S-1)

where double point ":" is the double-dot product operator, indicating the sum of total angular indices; the boldface symbols **T** and **S** are stress tensor and strain tensor, respectively; *c* and *η* are the stiffness coefficient matrix and viscosity coefficient matrix of the solid respectively.

For an isotropic solid medium, we have its stiffness matrix written as

$c=\left(\left.\begin{matrix} c_{11} & c_{12} & c_{12} & 0 & 0 & 0 \\ c_{12} & c_{11} & c_{12} & 0 & 0 & 0 \\ c_{12} & c_{12} & c_{11} & 0 & 0 & 0 \\ 0 & 0 & 0 & c_{44} & 0 & 0 \\ 0 & 0 & 0 & 0 & c_{44} & 0 \\ 0 & 0 & 0 & 0 & 0 & c_{44} \end{matrix}\right)\right.$ (S-2)

where *c*_11_ *= c*_12_ *+ 2c*_44_, for *c*_11_, *c*_12_, and *c*_44_, there are only two independent elements.

Analogous to the stiffness matrix of a solid medium, there is also a viscosity coefficient matrix corresponding to the stiffness coefficient matrix, which describes the viscosity of isotropic solid,

$\eta=\left(\left.\begin{matrix} \eta_{11} & \eta_{12} & \eta_{12} & 0 & 0 & 0 \\ \eta_{12} & \eta_{11} & \eta_{12} & 0 & 0 & 0 \\ \eta_{12} & \eta_{12} & \eta_{11} & 0 & 0 & 0 \\ 0 & 0 & 0 & \eta_{44} & 0 & 0 \\ 0 & 0 & 0 & 0 & \eta_{44} & 0 \\ 0 & 0 & 0 & 0 & 0 & \eta_{44} \end{matrix}\right)\right.$ (S-3)

A similar relationship exists between viscosity elements ($\eta_{11}\text{= }\eta_{12}\text{+ 2}\eta_{44}$), and only two independent variables exist for an isotropic solid's viscosity coefficient matrix elements.

It is worth noting that formula (S-1) considers both the elastic force in the "spring oscillator" model and the frictional force of the vibrating particle caused by the viscosity of the solid.

We use the strain and stiffness coefficient of solid medium to simulate the particle displacement and the spring's stubborn coefficient in the "physical model of spring oscillators" and use the derivative of strain concerning time and the viscosity coefficient matrix of viscous solid to analogy the moving speed of the particle (or tiny volume element) and the friction resistance. For the particle vibration corresponding to a longitudinal wave, the relationships between them are connected by scale factors ($a_{1}$, $a_{2}$ and $a_{3}$) as follows

$R_{m}=a_{1}\eta_{11}$ (S-4)

$m=a_{2}\rho$ (S-5)

$k_{c}=a_{3}c_{11}$ (S-6)

It is our next step work to determine the proportion factors ($a_{1}$, $a_{2}$ and $a_{3}$) for the actual rock strata experimentally.

**Reference**

[S1] Auld, B.A. Acoustic Fields and Waves in Solids. New York: John Wiley and Sons, 1973.
